# ^166^Holmium–^99^^m^Technetium dual-isotope imaging: scatter compensation and automatic healthy-liver segmentation for ^166^Holmium radioembolization dosimetry

**DOI:** 10.1186/s40658-022-00459-x

**Published:** 2022-04-21

**Authors:** Martina Stella, Arthur J. A. T. Braat, Marnix G. E. H. Lam, Hugo W. A. M. de Jong, Rob van Rooij

**Affiliations:** Department of Radiology and Nuclear Medicine, UMC Utrecht, Heidelberglaan 100, 3584 CX Utrecht, The Netherlands

**Keywords:** Radioembolization, ^166^Holmium, ^99m^Technetium, Dual isotope, Segmentation, Dosimetry, Healthy-liver

## Abstract

**Background:**

Partition modeling allows personalized activity calculation for holmium-166 (^166^Ho) radioembolization. However, it requires the definition of tumor and non-tumorous liver, by segmentation and registration of a separately acquired CT, which is time-consuming and prone to error. A protocol including ^166^Ho-scout, for treatment simulation, and technetium-99m (^99m^Tc) stannous phytate for healthy-liver delineation was proposed. This study assessed the accuracy of automatic healthy-liver segmentation using ^99m^Tc images derived from a phantom experiment. In addition, together with data from a patient study, the effect of different ^99m^Tc activities on the ^166^Ho-scout images was investigated. To reproduce a typical scout procedure, the liver compartment, including two tumors, of an anthropomorphic phantom was filled with 250 MBq of ^166^Ho-chloride, with a tumor to non-tumorous liver activity concentration ratio of 10. Eight SPECT/CT scans were acquired, with varying levels of ^99m^Tc added to the non-tumorous liver compartment (ranging from 25 to 126 MBq). For comparison, forty-two scans were performed in presence of only ^99m^Tc from 8 to 240 MBq. ^99m^Tc image quality was assessed by cold-sphere (tumor) contrast recovery coefficients. Automatic healthy-liver segmentation, obtained by thresholding ^99m^Tc images, was evaluated by recovered volume and Sørensen–Dice index. The impact of ^99m^Tc on ^166^Ho images and the role of the downscatter correction were evaluated on phantom scans and twenty-six patients’ scans by considering the reconstructed ^166^Ho count density in the healthy-liver.

**Results:**

All ^99m^Tc image reconstructions were found to be independent of the ^166^Ho activity present during the acquisition. In addition, cold-sphere contrast recovery coefficients were independent of ^99m^Tc activity. The segmented healthy-liver volume was recovered fully, independent of ^99m^Tc activity as well. The reconstructed ^166^Ho count density was not influenced by ^99m^Tc activity, as long as an adequate downscatter correction was applied.

**Conclusion:**

The ^99m^Tc image reconstructions of the phantom scans all performed equally well for the purpose of automatic healthy-liver segmentation, for activities down to 8 MBq. Furthermore, ^99m^Tc could be injected up to at least 126 MBq without compromising ^166^Ho image quality.

*Clinical trials* The clinical study mentioned is registered with Clinicaltrials.gov (NCT02067988) on February 20, 2014.

## Background

Holmium-166 (^166^Ho) radioembolization is an established treatment for liver malignancies [1]. The current clinical practice for ^166^Ho radioembolization includes pre-treatment planning and treatment, together with post-treatment verification. The pre-treatment phase can be performed using QuiremScout™ (Quirem Medical BV, Deventer, NL), particles which are shaped identical to the microspheres used for the treatment, ^166^Ho-microspheres (QuiremSpheres™, Quirem Medical BV, Deventer, NL). Using the ^166^Ho-microspheres for both procedures (pre-treatment and treatment) has the benefit of improving the intrahepatic distribution prediction in comparison with current clinical standard (technetium-99m macroaggregated albumin or ^99m^Tc-MAA) [2]. The activity distribution imaged in this phase serves as a predictor for the radiation dose distribution during the treatment and can be used to avert a potential extrahepatic deposition. In addition, it enables partition modeling [3] which allows a personalized activity calculation for ^166^Ho radioembolization. However, this requires segmentation of tumors and non-tumorous tissue on anatomical images, which is typically performed using a complementing contrast enhanced CT, usually acquired up to weeks before the treatment. Consequently, these segmented volumes of interest (VOIs), tumors and non-tumorous area, should be registered to the SPECT/CT to perform dosimetry. A similar workflow applies to the post-treatment phase, to assess treatment outcome and response. So far, manual segmentation and manual image registration are currently most commonly applied in clinical practice. These manual processes are time-consuming, prone to error and introduce inter-observer variability. Therefore, a protocol to automatically segment and register the VOIs would tackle these drawbacks, allowing an automatic workflow for planning and evaluation of the treatment. To this purpose, a dual-isotope protocol was suggested by Lam et al*.* [4]. It is based on ^166^Ho microspheres, which serve as treatment simulation, and technetium-99m (^99m^Tc) stannous phytate, which accumulates in the healthy-liver tissue and provides a healthy-liver demarcation (^166^Ho–^99m^Tc dual-isotope protocol). These two compounds can be imaged simultaneously with a single SPECT acquisition and then be reconstructed into two images: ^166^Ho and ^99m^Tc, avoiding any registration procedure.

**Fig. 1:**
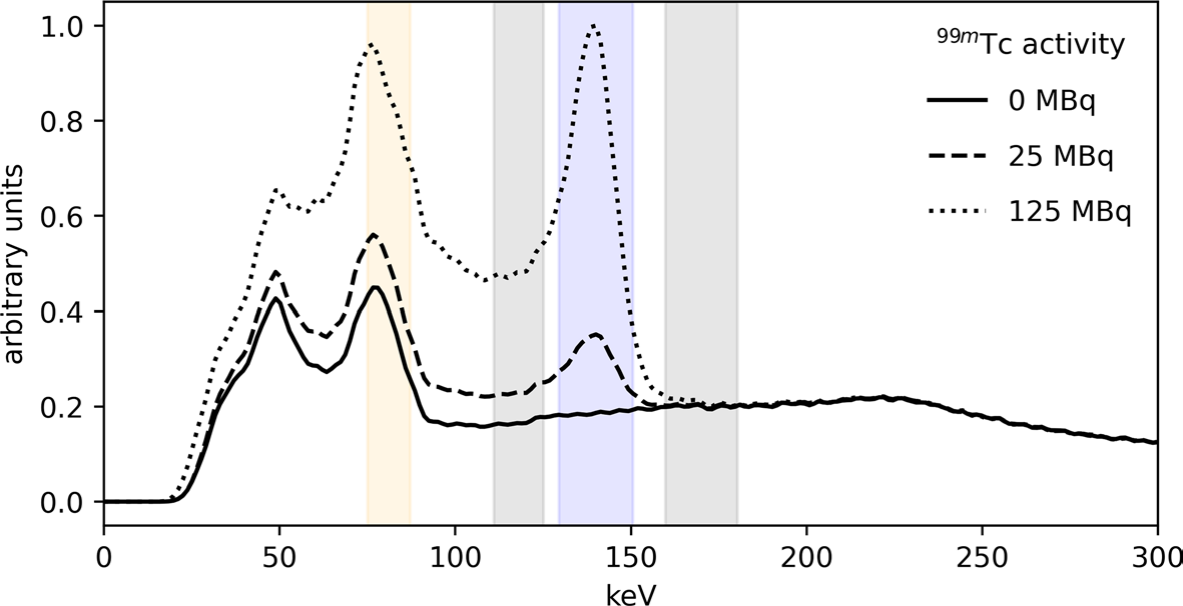
Recorded ^166^Ho–^99m^Tc spectra. Spectra recorded by the SPECT scanner of an anthropomorphic phantom filled with ^166^Ho and ^99m^Tc. Curves are depicted for ^166^Ho:^99m^Tc activity combinations (in MBq) of: 282:0 (solid line), 268:25 (dashed line), and 250:125 (dotted line). The curves were scaled such that the tails of the spectra overlap for visual comparison. The relative difference between the spectra is attributed to ^99m^Tc. Orange and blue areas represent the ^166^Ho and ^99m^Tc photopeak windows, respectively, while the gray areas depict the scatter windows (see Table 2)

However, the presence of the two radionuclides leads to a reciprocal influence between the two (depicted in Fig. 1), which has to be taken into account during the image reconstruction phase. In particular, the ^166^Ho main photopeak, at 81 keV, is affected by the downscatter from ^99m^Tc, which has its main photopeak at 140 keV. Vice versa, ^99m^Tc is contaminated by downscatter from the ^166^Ho high-energy gamma emissions and bremsstrahlung.

The ^166^Ho–^99m^Tc dual-isotope protocol is currently implemented in our institute using 50 MBq of ^99m^Tc administered right after 250 MBq ^166^Ho scout, leading to a ^166^Ho:^99m^Tc activity ratio of 5. The decision to use this particular ratio was based on the results of a phantom study together with visual interpretation and consensus reading by two nuclear medicine physicians and a medical physicist [5]. Within the clinical practice, ^166^Ho–^99m^Tc dual-isotope scans are reconstructed using 3D OSEM algorithm (Flash 3D; Siemens). The impact of this protocol on ^166^Ho image quality was investigated through a comparison between patient scans, acquired before and after the additional ^99m^Tc injection [6].

This current study expands on the previous work by van Rooij et al*.* [5] who used a Monte Carlo-based reconstruction method to correct for the crosstalk interactions between the two isotopes. However, because this reconstruction method is not available for everyday clinical practice, the current study focuses on the applicability of the ^166^Ho–^99m^Tc dual-isotope protocol in the clinical scenario, using commercially available reconstruction software. The impact of different ^99m^Tc activities on the ^166^Ho-scout image quality and, vice versa, the impact of ^166^Ho scout on ^99m^Tc image quality and the accuracy of healthy-liver segmentation were investigated using scans reconstructed according to the clinical protocol. To understand the impact of ^99m^Tc on ^166^Ho image quality and its associated effect on downscatter correction during image reconstruction, ^166^Ho image quality has been assessed by multiple metrics. Image uniformity, contrast recovery coefficients and ^166^Ho count density, i.e., the voxel value in ^166^Ho reconstructions, were measured for various amounts of ^99m^Tc activity, investigating their dependency on the k-factor used for ^166^Ho image reconstruction. This will make the ^166^Ho–^99m^Tc dual-isotope protocol more suitable for its adoption in ^166^Ho radioembolization practice, independent of the treatment institution.

## Materials and methods

To investigate ^99m^Tc image reconstructions for the purpose of healthy-liver segmentation during a ^166^Ho-scout procedure, an anthropomorphic phantom using various concentrations of ^99m^Tc in the healthy-liver compartment in presence or not of ^166^Ho was used. To mimic patient tumor/liver uptake, ^166^Ho was injected in the healthy-liver compartment. Two ^166^Ho-filled spheres with a higher concentration resembling tumors with a high uptake were placed within the healthy-liver compartment. ^99m^Tc was added only to the healthy-liver compartment and not to the tumors, simulating the expected distribution of ^99m^Tc stannous phytate. For the ^166^Ho–^99m^Tc dual-isotope study, the ^166^Ho activity was held constant at 250 MBq (as per scout prescription), while ^99m^Tc was varied across a range of activities.

^99m^Tc image quality for varying levels of ^99m^Tc activity was evaluated though several metrics: image uniformity, cold-sphere contrast recovery and the accuracy of healthy-liver segmentations (obtained by thresholding). For comparison, these acquisitions were repeated without ^166^Ho in the phantom in order to investigate the influence of ^166^Ho on ^99m^Tc reconstructions.

The effect that the addition of ^99m^Tc has on ^166^Ho images was investigated by comparing ^166^Ho reconstructions from similar scans, performed with various amounts of ^99m^Tc. Specifically, ^166^Ho count densities (the voxel values in the reconstructions) in the healthy-liver compartment were compared between reconstructions. To this end, phantom measurements and multiple patient scans were analyzed.

Details regarding the measurements and the corresponding metrics are summarized in Table1

**Table 1:** Summary of the experiments

	Protocol	^166^Ho [MBq]	^99m^Tc [MBq]	Scans	Reconstruction	Aim	Metric
Phantom	^166^Ho–^99m^Tc dual isotope	250	25–126	8	^166^Ho	Uniformity	Coefficient of variation
						Contrast	Hot-sphere contrast recovery
						Downscatter	^166^Ho count density
					^99m^Tc	Uniformity	Coefficient of variation
						Contrast	Cold-sphere contrast recovery
						Segmentation	Recovered volume Sørensen-Dice index
	^99m^Tc only	0	8–240	42	^99m^Tc	Uniformity	Coefficient of variation
						Contrast	Cold-sphere contrast recovery
						Segmentation	Recovered volume
							Sørensen–Dice index
Patients	^166^Ho–^99m^Tc dual isotope	224 (35)^a^	50	26	^166^Ho	Downscatter	^166^Ho count density difference
	^166^Ho-only	224 (35)^a^	0	26	^166^Ho		

^a^Median (and interquartile range) of administered activity among patient scout procedures

## Phantom

### Phantom characteristics

An anthropomorphic phantom (model ECT/TOR/P), including lungs and liver, was used to mimic patient anatomy. Two fillable spheres (S1 and S2) were placed in the liver to resemble tumors of different sizes.

For the ^166^Ho–^99m^Tc dual-isotope measurements, the healthy-liver compartment (1205 mL) and spheres (S1: volume = 24.2 mL, radius = 1.79 cm and S2: volume = 15.7 mL and radius = 1.55 cm) were filled with ^166^Ho-chloride with a sphere to healthy-liver compartment concentration ratio of 10:1, resembling a high tumor-to-non-tumor uptake, typically reported for large, highly vascularized tumors [7, 8]. This resulted in an activity percentage, with respect to the total activity in the liver, equal to 15.10% for S1 and 9.76% for S2. In order to consistently achieve an equivalent of 250 MBq of ^166^Ho across the various measurements, which resembles the prescribed scout activity as used in the clinical studies [9], the imaging time for each scan was adjusted (17.8–38.2 s per projection) with respect to the clinical protocol (20 s per projection) to compensate for ^166^Ho decay between scans. The validity of this approach was based on the assumption that the difference in dead time had little effect on count statistics. The measured dead time rate shifted on average from 4.5% at the higher activities to 2.5% at lower activities compensated by longer scanning time. ^99m^Tc activity was injected multiple times in the healthy-liver compartment leading to various ^166^Ho:^99m^Tc ratios, ranging from 2 to 10. Effective ^99m^Tc activity, modified to correct for the varying imaging times, ranged from 25 to 126 MBq.

In a separate series of measurements, the anthropomorphic phantom was filled only with ^99m^Tc in the healthy-liver compartment (activity ranging from 8 to 240 MBq). These ^99m^Tc-only measurements were used as reference for comparison of the ^99m^Tc images acquired in presence of ^166^Ho.

No radioactivity was injected into the lung compartment of the phantom, nor the torso compartment.

### Phantom data acquisition

All images were obtained using a Symbia T SPECT/CT scanner (Siemens, Erlangen, Germany), using medium-energy collimators. Projections were recorded on a 128 × 128 matrix (pixel spacing, 4.8 × 4.8 mm), with 120 angles, over a non-circular 360° orbit using step-and-shoot mode. Energy windows used for image acquisition are summarized in Table2

**Table 2:** Energy window characteristics

Window name	Center of the energy window	Width of the energy window (%)
^166^Ho photopeak	81 keV	15
Scatter__118_	118 keV	12
^99m^Tc photopeak	140 keV	15
Scatter__170_	170 keV	12

### Phantom data reconstruction

All images were reconstructed using commercially available software (Siemens Flash3D), with 10 iterations, 8 subsets, incorporating scatter and attenuation correction. No post-reconstruction filtering was applied.

#### ^166^Ho-^99m^Tc dual-isotope downscatter correction

^166^Ho images were reconstructed with window-based scatter correction, using projections acquired in the 118 keV energy window (scaled by a k-factor) as an estimate for downscatter in the 81 keV photopeak window originating from both ^99m^Tc and higher-energy ^166^Ho gamma emissions and bremsstrahlung.

Starting from the k-factor value previously computed by dividing the counts in the 81 keV and 118 keV energy window of ^166^Ho–^99m^Tc dual-isotope projections [6], the k-factor for different ^99m^Tc activities was empirically investigated by reconstructing ^166^Ho images for a variety of k-factors ranging from 0.65 to 1.30 with a 0.05 interval. The optimal value of the k-factor was tuned by measuring, and minimizing, the impact of ^99m^Tc activity on the ^166^Ho count density measured on ^166^Ho image reconstruction. Photopeak scatter, i.e., scattered photons originating from the 81 keV ^166^Ho photopeak, was not accounted for.

^99m^Tc images were reconstructed using the 118 keV and 170 keV windows for triple-energy-window scatter correction, and the scatter was estimated as:

$${S}_{E}= \left(\frac{{C}_{L}}{{W}_{L}}+\frac{{C}_{U}}{{W}_{U}}\right)\times \frac{1}{2}\times {W}_{PP}=\left(\frac{{W}_{PP}}{{W}_{L}\times 2}\right)\times {C}_{L}+\left(\frac{{W}_{PP}}{{W}_{U}\times 2}\right)\times {C}_{U}$$

where ${C}_{L}$ and ${C}_{U}$ are the recorded projections for the lower (Scatter__118_) and upper scatter (Scatter__170_) windows, respectively, and ${W}_{L}$, ${W}_{U}$ and ${W}_{PP}$ are the widths of the lower, upper and main photopeak energy windows.

For consistency, this method was also applied when no ^166^Ho activity was present in the phantom.

### Phantom data analysis

#### VOI definition

VOIs matching the phantoms’ liver compartment and sphere inserts were defined on a high resolution CT. The sphere VOIs were subtracted from the liver mask to produce the healthy-liver compartment VOI. These pre-defined VOIs were registered to each SPECT/CT reconstruction using Elastix [10, 11]. Grid matrices were super-sampled to allow partial voxels to be included within the VOIs.

#### Uniformity

The healthy-liver uniformity for different ^99m^Tc activities was quantified by the coefficient of variation (COV), defined as the ratio of the standard deviation to the mean, computed within the healthy-liver compartment VOI, for both ^166^Ho and ^99m^Tc reconstructions. The COV was computed for each ^99m^Tc image, acquired either in presence or not of ^166^Ho in the phantom. A binary erosion of 1 cm [12] on the healthy-liver mask was applied to avoid edge effects.

#### Contrast recovery

Image quality can be assessed by analyzing the contrast recovery coefficient for either hot or cold spheres (${Q}_{H}$ or ${Q}_{C},$ respectively), generally defined as:

$$Q = \frac{{C_{S} /C_{B} - 1}}{{(R - 1)}} \times 100\%$$

where ${C}_{S}$ is the mean intensity measured in the sphere VOI, ${C}_{B}$ is the mean intensity measured in the healthy-liver compartment VOI, and R is the nominal activity concentration ratio between spheres and healthy-liver compartment. However, for cold spheres, R is zero by definition.

The effect of adding ^99m^Tc activity to the healthy-liver compartment was assessed by measuring the contrast recovery coefficients on both ^166^Ho and ^99m^Tc reconstructions (${Q}_{H}$ or ${Q}_{C},$ respectively). The nominal activity concentration ratio between spheres and healthy-liver compartment, R, was 10 for ^166^Ho reconstructions, but since only ^166^Ho was present in the spheres, and not ^99m^Tc, R was zero for all ^99m^Tc reconstructions.

#### Healthy-liver segmentation

The usability of the ^99m^Tc scans for the purpose of automatic segmentation of the healthy-liver was investigated by analyzing the overlap between the segmentations and the pre-defined healthy-liver compartment VOI for images acquired at different ^99m^Tc activities. The segmentations were obtained using a thresholding procedure. The accuracy of a standard thresholding procedure relies on the choice of the threshold value, which is typically defined as a percentage of the maximum image intensity. This, however, implies that the segmentation relies on a single voxel value, the maximum, which is prone to inaccuracy due to noise. To reduce this dependency, the threshold value was instead based on a percentage (α) of the maximum value after having smoothened the image using a 3D Gaussian filter. The threshold was then applied back to the original, un-smoothened, image to produce the segmentation.

For every individual scan, an optimal threshold percentage α could be determined by applying an optimization routine which varied α to correctly recover the volume of the healthy-liver in the phantom. However, as these values for α may be different between scans, a single value to apply to all scans was defined as the average of all individual optimal values.

The accuracy of the healthy-liver segmentation using ^99m^Tc images was evaluated by assessing both the recovered healthy-liver compartment and the resulting cold spheres (i.e., the tumors). The ratio between the segmented volumes and the nominal volumes was computed for the three VOIs (cold sphere S1 and S2, and healthy-liver compartment). In addition, the overlap between the segmentations and the nominal VOIs was assessed through the Sørensen–Dice index [13].

#### Statistical analysis

For the above-mentioned metrics (uniformity, cold-sphere contrast recovery coefficients and healthy-liver segmentation), a t test was used to determine if there was a significant difference between the measurements acquired in presence or not of ^166^Ho. P-values were reported only if a statistically significant difference was found.

#### Impact of k-factor on ^166^Ho phantom reconstructions

SPECT reconstructions of ^166^Ho images suffer from downscatter induced by higher-energy gamma emissions and bremsstrahlung, detected in the 81 keV ^166^Ho photopeak window. The 118 keV energy window, scaled with a k-factor, is used as an estimate for these downscatter contributions. In case of dual-isotope ^166^Ho–^99m^Tc imaging, there is an additional downscatter contribution arising from the ^99m^Tc photopeak at 140 keV. Ideally, however, with a well-chosen k-factor, the ^166^Ho reconstructions are independent of ^99m^Tc activity.

The impact of ^99m^Tc on ^166^Ho images can be assessed by the COV and the contrast recovery coefficients (of hot spheres), similarly to the ^99m^Tc analysis. However, both these metrics strongly depend on the ^166^Ho count density in the healthy-liver compartment.

To determine the optimal k-factor, the count density in the healthy-liver compartment VOI was measured for all ^166^Ho images, reconstructed for a range of k-factors (0.65–1.30 with a 0.05 step interval). For each k-factor, the relative change in ^166^Ho count density was determined as a function of ^99m^Tc activity.

### Patient data

To clinically evaluate the findings regarding the k-factor obtained using the phantom scans, a similar analysis was applied to images from patient procedures, for which both a ^166^Ho–^99m^Tc dual-isotope and a ^166^Ho-only acquisition was available.

For all patient SPECT/CT acquisitions used in this study, informed consent was obtained as part of the HEPAR PLuS study [14]. Twenty-six scout (pre-treatment) procedures performed on patients with liver metastases of neuroendocrine tumors were analyzed (median administered activity (and interquartile range): 224 (35) MBq of ^166^Ho).

According to the HEPAR PLuS study protocol, for each scout procedure, two SPECT/CT images were acquired: a ^166^Ho-only SPECT/CT and, after administration of ^99m^Tc-stannous phytate, a ^166^Ho dual-isotope SPECT/CT. All scans were acquired and reconstructed using the same protocols as those adopted for the ^166^Ho–^99m^Tc dual-isotope phantom data. Similar to the ^166^Ho phantom scans, ^166^Ho patient images were reconstructed using multiple k-factors ranging from 0.65 to 1.30 with step 0.05.

To assess the impact of the k-factor on ^166^Ho patient reconstructions, a volume of interest was defined containing the healthy-liver (by thresholding the ^99m^Tc image). Within this healthy-liver VOI, the ^166^Ho count density was determined for both acquisitions, ^166^Ho-only and ^166^Ho dual isotope, using the same k-factor for both reconstructions. The percentage difference in ^166^Ho count density between the ^166^Ho dual isotope and ^166^Ho-only acquisition was computed for each k-factor (ranging from 0.65 to 1.30).

## Results

### Phantom

#### Uniformity

**Fig. 2:**
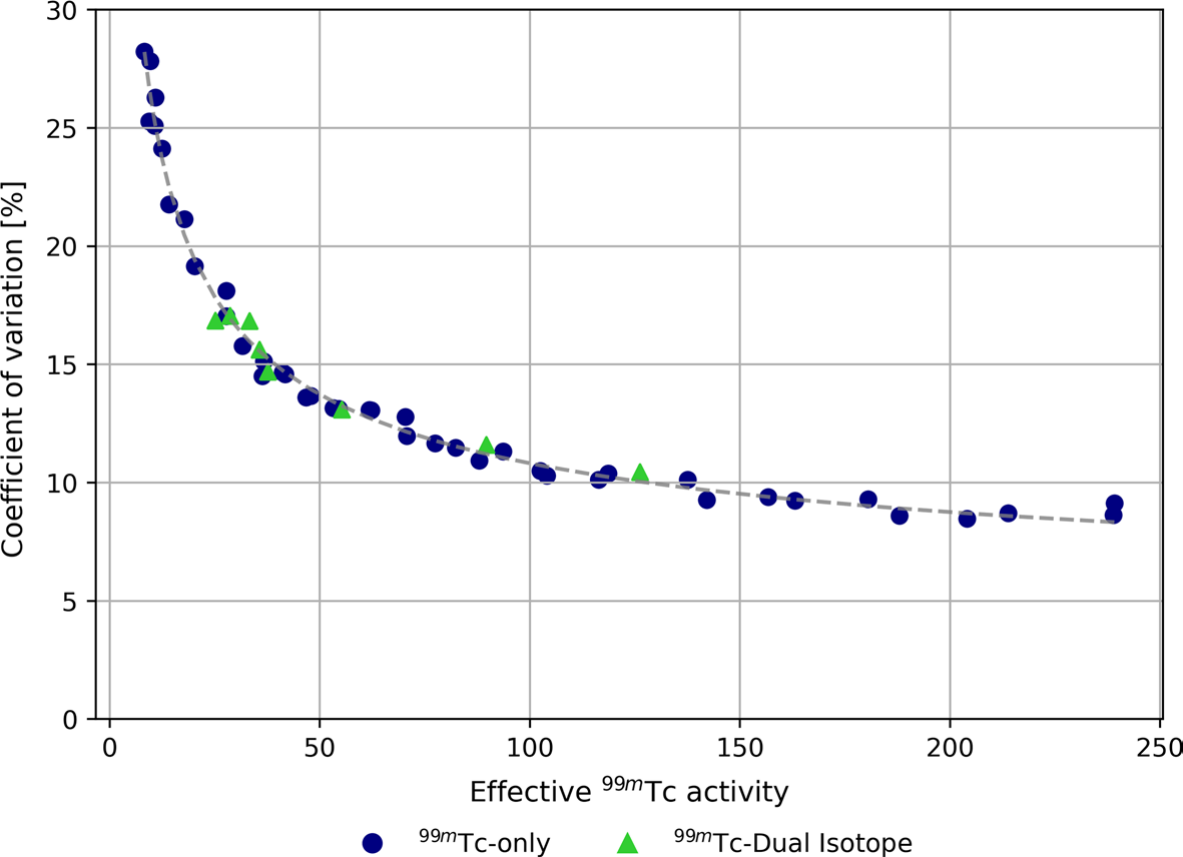
Coefficient of variation computed on the ^99m^Tc images within the healthy-liver compartment VOI. The coefficient of variation, measuring the level of inhomogeneity in the healthy-liver compartment VOI, is displayed as function of effective ^99m^Tc activity in the phantom. Blue circles refer to measurements acquired with ^99m^Tc only, while green triangles represent measurements acquired with ^99m^Tc in presence of ^166^Ho (dual isotope). The COV decreases with the square root of ^99m^Tc activity, down to an offset which is assumed be present due to limited imaging resolution (independent of ^99m^Tc activity). The gray dashed line depicts a fit to the data, where the offset was found to be 3.8%

The ^99m^Tc image uniformity, measured as the coefficient of variation within the healthy-liver compartment, is displayed in Fig. 2. The COV decreased with the square root of ^99m^Tc activity, but was independent on the presence of 250 MBq of ^166^Ho in the phantom. No statistically significant difference was found between ^99m^Tc images acquired in presence or not of ^166^Ho.

The ^99m^Tc image uniformity, measured as the coefficient of variation within the healthy-liver compartment, is displayed in Fig. 2. The COV decreased with the square root of ^99m^Tc activity, converging to a minimum of approximately 3.8% (likely limited by the imaging resolution), but was independent on the presence of 250 MBq of ^166^Ho in the phantom. No statistically significant difference was found between ^99m^Tc images acquired in presence or not of ^166^Ho.

#### Contrast recovery of cold spheres

**Fig. 3:**
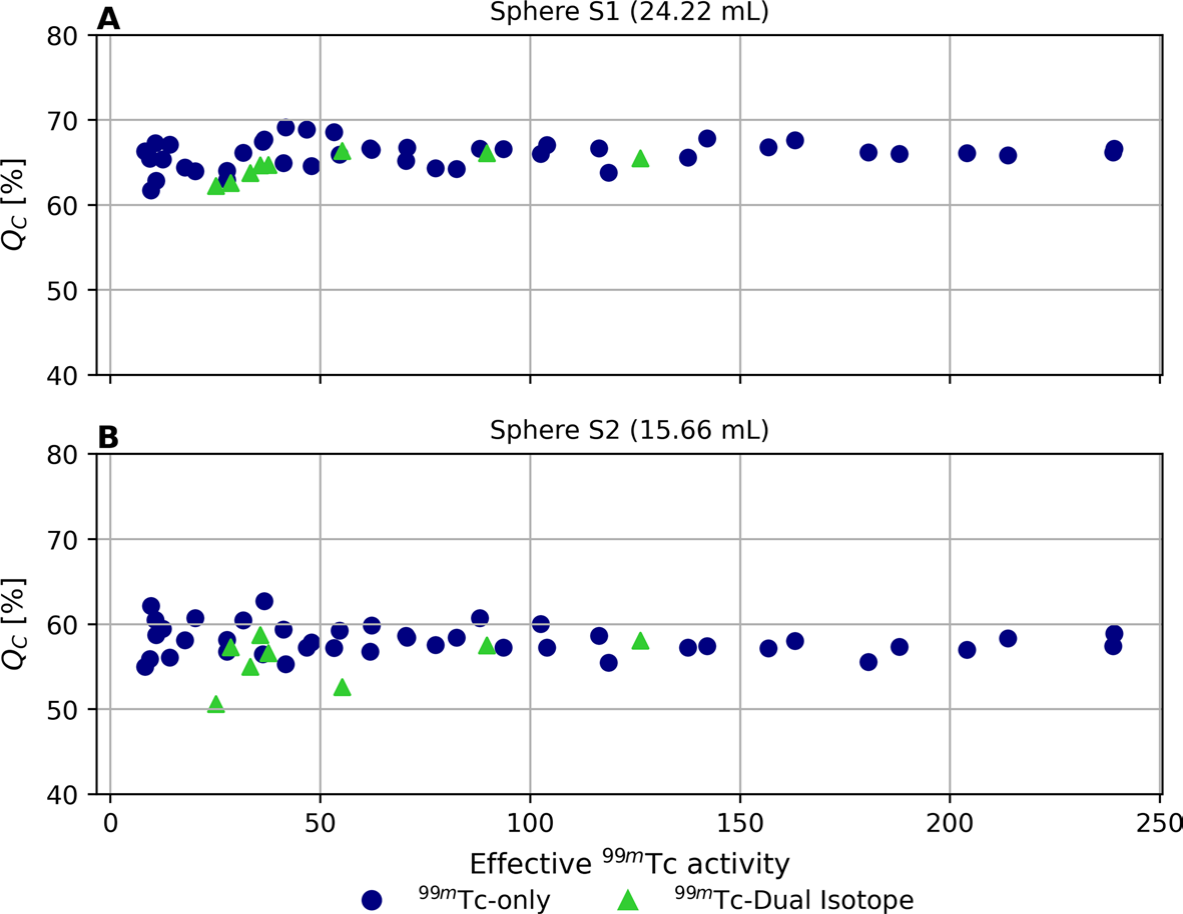
Contrast recovery coefficient of cold spheres (${Q}_{c}$) as function of total effective ^99m^Tc activity. ${Q}_{c}$ computed on ^99m^Tc images acquired in presence of different ^99m^Tc activities. Results are displayed as function of the total effective ^99m^Tc activity in the phantom. Panel **A** refers to cold sphere S1 and panel **B** to cold sphere S2. Blue circles refer to measurements acquired in presence of ^99m^Tc only, while green triangles represent measurements acquired in presence of both ^99m^Tc and ^166^Ho

Cold-sphere contrast recovery coefficients as function of effective ^99m^Tc activity in the phantom are reported in Fig. 3**A**, **B**, for sphere S1 and sphere S2, respectively. Mean ± standard deviation of ${Q}_{C}$ was 65.7% ± 1.6% for sphere S1 and 57.8% ± 2.1% for sphere S2. At low ^99m^Tc activities (< 50 MBq), a higher spread in ${Q}_{C}$ (Fig. 3) was visually noticeable. No statistically significant difference was found between ^99m^Tc images acquired either in presence or not of ^166^Ho.

#### Usability of ^99m^Tc images for automatic healthy-liver segmentation

The procedure to determine the optimal threshold percentage α was repeated for a range of Gaussian filter sizes, where a width (sigma) of 15 mm was found to produce the smallest variance among segmented volumes. For this filter size, the averaged optimal threshold percentage α adopted for the automatic segmentation process was 40%.

**Fig. 4:**
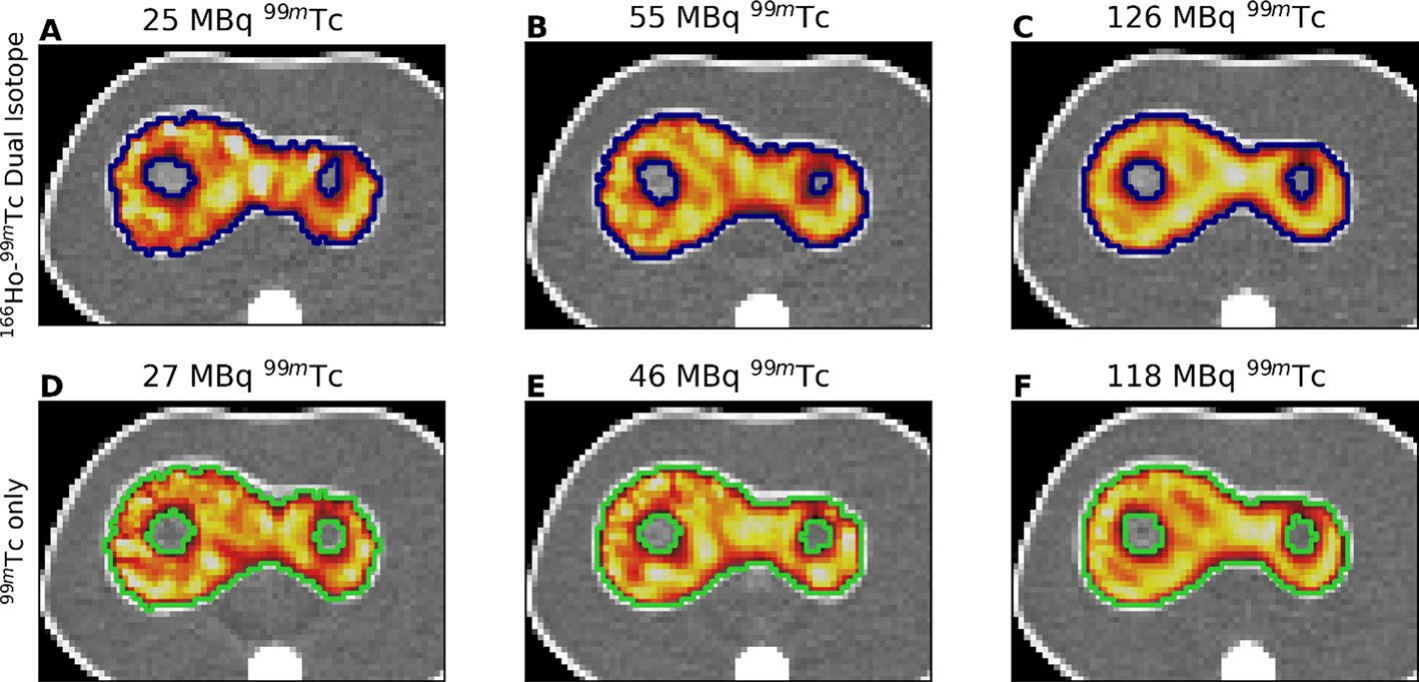
Axial view of ^99m^Tc reconstructions and healthy-liver segmentations. Healthy-liver segmentations of ^99m^Tc SPECT/CTs, acquired in presence of 250 MBq^166^Ho (top panel) and without ^166^Ho (bottom panel). Three ^99m^Tc activity levels are presented to indicate the effect on image quality and segmentation accuracy

Figure 4 shows the axial view of six phantom SPECT/CT scans with the healthy-liver contour overlapped resulting from the automatic segmentation process.

**Fig. 5:**
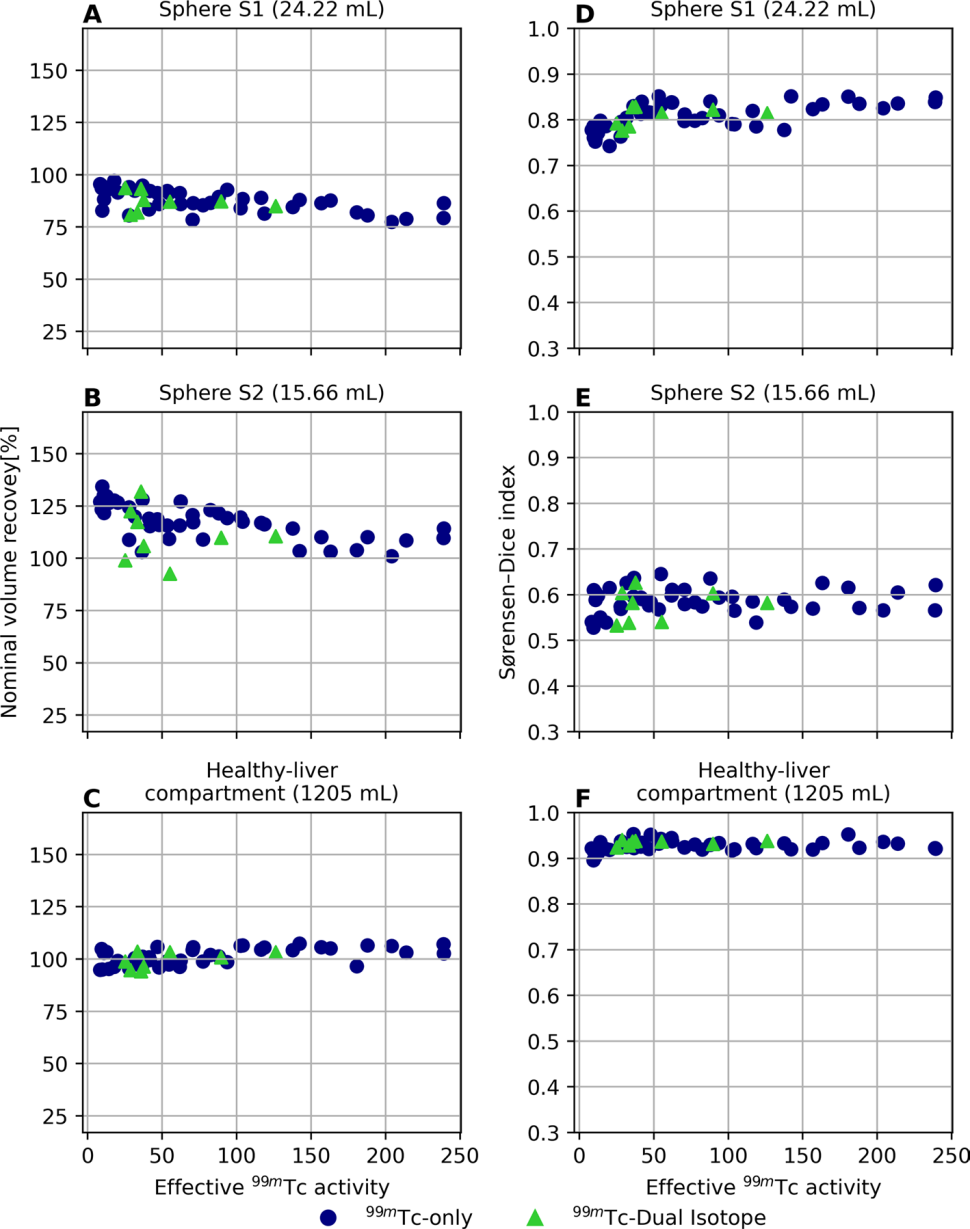
Nominal volume recovery and Sørensen–Dice index obtained from segmentation of the ^99m^Tc images using a threshold approach. On the left, the percentage of recovered nominal volume using the threshold approach for the three VOIs under investigation is shown: sphere S1 (panel **A)**, sphere S2 (panel **B**) and healthy-liver compartment (panel **C**). Blue circles refer to measurements acquired in presence of ^99m^Tc only, while green triangles represent measurements acquired in presence of both ^99m^Tc and ^166^Ho. The results are displayed as function of the total effective ^99m^Tc activity in the phantom. On the right (panel **D, E** and **F**), the corresponding Sørensen–Dice index is shown

Results for the volume recovery percentage and Sørensen–Dice index as function of the effective ^99m^Tc activity are depicted in Fig. 5**A**, **D** which refer to cold sphere S1, **B** and **E** to cold sphere S2 and panel **C** and **F** to the healthy-liver compartment. Mean ± standard deviation for the volume recovery percentage was 88.8% ± 5.3%, 118.2% ± 12.0% and 99.6% ± 3.8% for cold sphere S1, S2 and healthy-liver compartment, respectively. Mean ± standard deviation for the Sørensen–Dice index was 0.79 ± 0.02, 0.58 ± 0.03 and 0.93 ± 0.01 for cold sphere S1, S2 and healthy-liver compartment, respectively. No statistically significant difference was found between ^99m^Tc images acquired either in presence or not of ^166^Ho.

#### Impact of k-factor on ^166^Ho phantom reconstructions

**Fig. 6:**
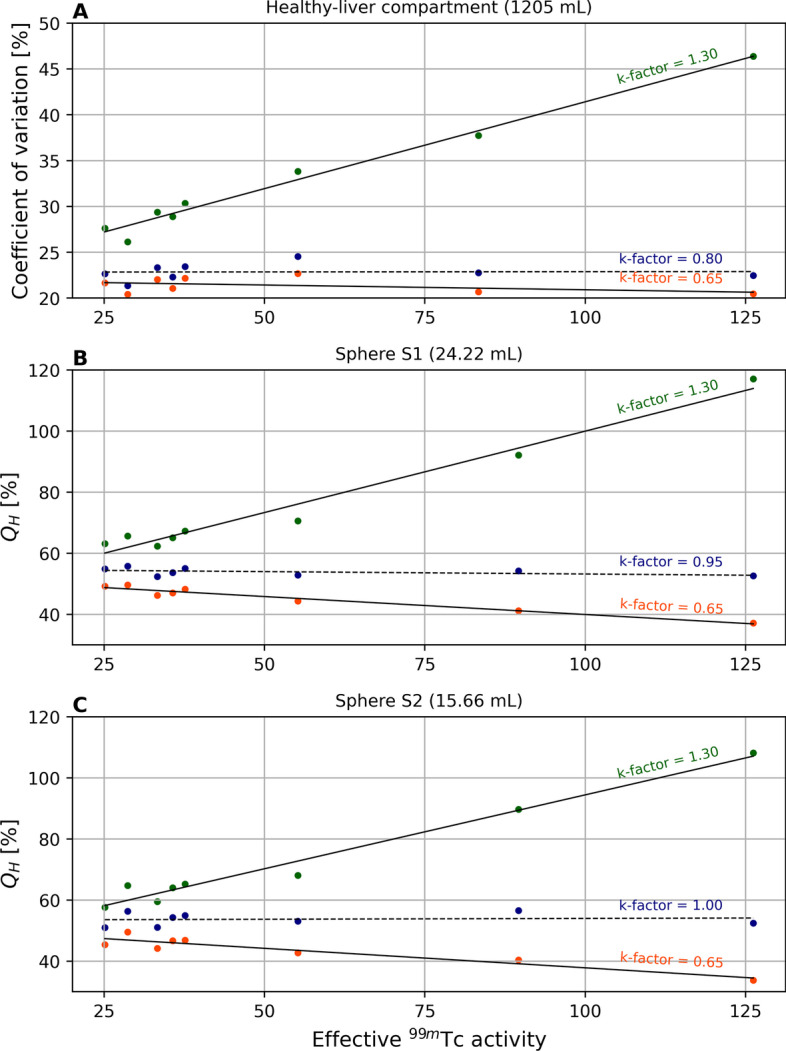
Quality analysis for the ^166^Ho images, reconstructed using several k-factors, as function of the ^99m^Tc activity in the phantom during the ^166^Ho–^99m^Tc dual-isotope acquisition. Coefficient of variation and contrast recovery coefficients for hot sphere S1 and S2 are depicted as function of effective ^99m^Tc activity in the phantom, for ^166^Ho reconstructions using three different k-factors. Panel **A** shows the coefficient of variation, measuring the level of inhomogeneity, computed on the ^166^Ho images within the healthy-liver compartment VOI. Panels** B** and **C** depict the contrast recovery coefficients for sphere S1 and S2, respectively. Ideally, these image-quality metrics for ^166^Ho are independent of ^99m^Tc activity if scatter from ^99m^Tc is sufficiently corrected for. The impact of the choice of k-factor can be observed by the dependence on ^99m^Tc activity, causing an offset of counts in the phantom background. The depicted k-factors are the lowest, the highest and the k-factor leading to the lowest dependency on ^99m^Tc for the considered metric

The coefficient of variation and the contrast recovery coefficients for the hot spheres, as function of the effective ^99m^Tc activity in the phantom, are depicted in Fig. 6 for several k-factors.

A poor choice for the k-factor will under- or over-correct ^99m^Tc scatter in the ^166^Ho image, causing an increase or decrease in apparent ^166^Ho signal in the phantom, approximately linear with ^99m^Tc activity. This dependency is illustrated in Fig. 6 where results are shown for the lowest and the highest k-factor. However, tuning the k-factor can reduce the impact of ^99m^Tc, as shown by the dashed lines in Fig. 6.

**Fig. 7:**
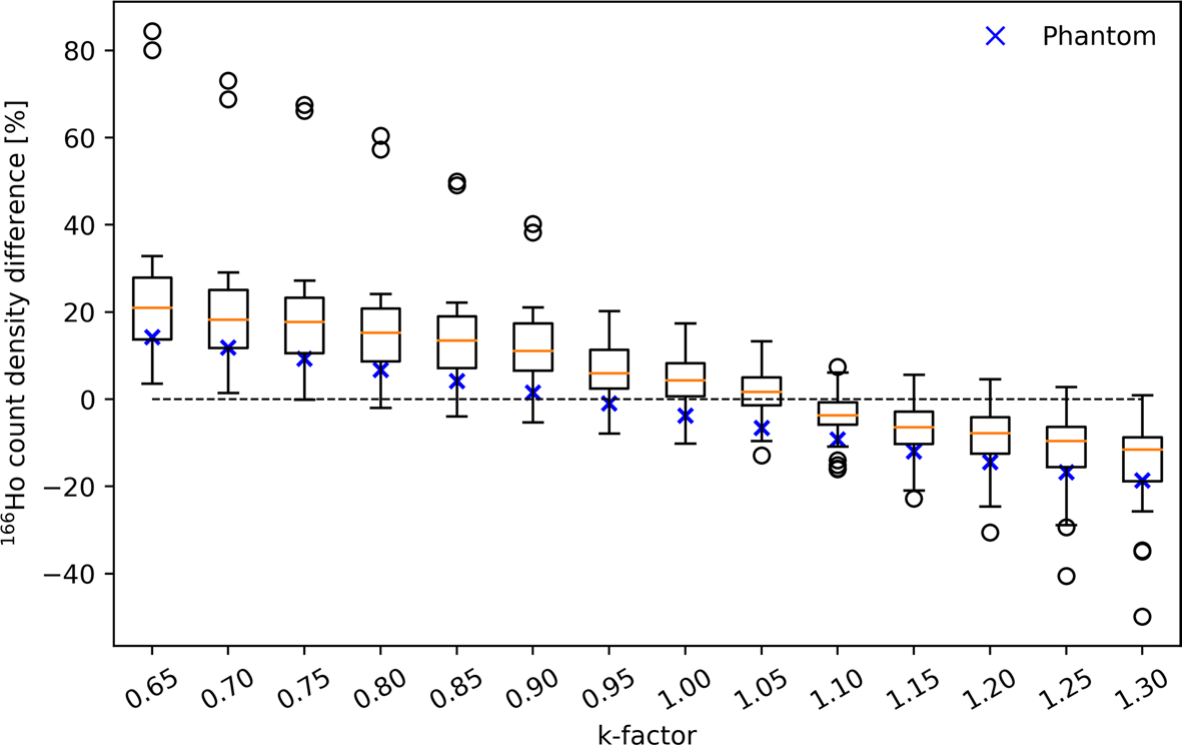
Dependence of ^99m^Tc scatter correction on ^166^Ho reconstructions. Scatter contributions from ^99m^Tc on ^166^Ho image reconstructions are corrected for using a dual-energy-window scatter correction. The scatter is estimated from the 118-keV energy window, positioned in between the ^166^Ho and ^99m^Tc photopeak, scaled by a k-factor. This graph shows the measured count density difference in ^166^ Ho reconstructions from ^166^Ho to ^99m^Tc dual-isotope acquisitions relative to ^166^Ho-only acquisitions, as function of the k-factor. Each boxplot represents the results for the 26 patient procedures. Count density was evaluated within the healthy-liver volume. Outlier values were due to a higher-than-average ^166^Ho concentration in the liver (due to either higher activity or relatively small liver volume). The blue crosses show the equivalent metric for the phantom study, where the count density difference was evaluated for a ^99m^Tc activity of 50 MBq

The percentage error in ^166^Ho count density, as a result of adding ^99m^Tc (evaluated at 50 MBq for consistency with the current clinical protocol), is plotted as a function of k-factor in Fig. 7 (blue crosses) along with the results from patient scans. It ranged from 14.3 to − 18.6%. The k-factor for which the ^166^Ho count density in the phantom was least dependent of ^99m^Tc activity was 0.95.

### Patient data

#### Impact of k-factor on ^166^Ho patient reconstructions

For the patient scans, the count density in ^166^Ho reconstructions was measured in the healthy-liver volume, for both ^166^Ho-only scans and ^166^Ho dual-isotope scans. The percentage difference between these measurements, attributed to downscatter from the additional ^99m^Tc in the liver, is plotted in Fig. 7 as a function of k-factor. For every k-factor, the data of the 26 patient procedures are summarized in a boxplot. A k-factor of 1.05 resulted in the smallest overall impact of ^99m^Tc on ^166^Ho image reconstructions with a median percentage difference (and interquartile range) of 1.7% (6.4%), ranging from -12.9% to 13.3% (Tables 1, 2).

## Discussions

Accurate image segmentation and registration are paramount to provide personalized dosimetry to radioembolization patients. However, these tasks are time-consuming and user-dependent, being currently performed manually.

The registration step could be avoided by adding a contrast enhanced CT acquisition to the SPECT/CT procedure. However, some limitations prevent this approach to be used in clinical practice. Typically, a contrast enhanced CT is already acquired prior to the scout procedure. Additionally, many SPECT/CT systems lack a contrast injector. Moreover, unless an algorithm for liver and tumor delineation on CT is clinically available, this approach would still require the manual segmentation of the volumes of interest.

The ^166^Ho-^99m^Tc dual-isotope protocol is a viable option to provide an automatic segmentation of the healthy-liver within the ^166^Ho radioembolization context. This study demonstrates that the reciprocal interaction between the two isotopes can mostly be negated, provided a proper k-factor for downscatter correction is applied for ^166^Ho reconstructions and a triple-energy-window scatter correction is applied for ^99m^Tc reconstructions.

### ^99m^Tc reconstructions

From the measurements performed using an anthropomorphic phantom, it was shown that ^99m^Tc image uniformity improved only little for higher ^99m^Tc activities (> 100 MBq), but that it was independent of ^166^Ho activity in the phantom. On average, the contrast recovery coefficients of the cold spheres were not dependent on ^99m^Tc activity, regardless of the presence of ^166^Ho. Nonetheless at lower ^99m^Tc activities (< 50 MBq) a higher spread for ${Q}_{C}$ was found (Fig. 2). The partial contrast recovery can be attributed to spill-in from activity in the healthy-liver compartment due to the limited SPECT system resolution.

Within the investigated range, the amount of ^99m^Tc activity did not have a significant effect on the accuracy of the healthy-liver segmentations. Using the same method for each segmentation, irrespective of ^99m^Tc activity or in presence or not of ^166^Ho, the healthy-liver compartment was recovered to 100% of its nominal volume, with a Sørensen–Dice index > 0.9. However, the cold spheres within the liver (representing tumors), were segmented to a lower accuracy as was evident from the reduced Sørensen–Dice indices.

### ^166^Ho reconstructions

For phantom images acquired at an effective ^166^Ho activity of 250 MBq in presence of ^99m^Tc, a major role was played by the choice of k-factor used for scatter correction, particularly for increasing ^99m^Tc activities. When reconstructed with adequate k-factors, the coefficient of variation, contrast recovery coefficients for the hot spheres and count density in the ^166^Ho main photopeak were, on average, not dependent on effective ^99m^Tc activity in the phantom. However, there is no single k-factor which is optimal to fully compensate for scatter across the whole ^166^Ho image. This emphasizes how the application of window-based scatter correction is only an approximation to correct a complex, nonlinear, phenomenon.

Patient scans showed the same k-factor dependence as demonstrated in the phantom study. Earlier work resulted in a k-factor of 1.15, which was based on simulations for ^166^Ho (by ignoring the 81-keV emission line) and by comparing counts in the projection windows for ^99m^Tc. In the current work, however, the effect of the k-factor used to correct for ^99m^Tc downscatter was measured directly on the resulting ^166^Ho reconstructions by considering the count density in the healthy-liver compartment (similar to patient dosimetry) depending on the presence of ^99m^Tc. A combined analysis of all patient and phantom scans (shown in Fig. 7) indicated that a k-factor of 1.05 resulted in the lowest impact of ^99m^Tc on ^166^Ho reconstructions overall.

The direct effect of inadequate scatter correction on ^166^Ho image reconstruction is an over- or under estimation of the count density, primarily in the healthy-liver compartment (due to the presence of ^99m^Tc stannous phytate). In clinical practice, dosimetry is often performed by first scaling the ^166^Ho scout image such that the total of counts in the image (including tumor uptake and extrahepatic depositions) corresponds to the planned therapeutic activity. Therefore, due to this scaling procedure, the impact of scatter on healthy-liver dosimetry is dependent on the relative activity distribution amongst the compartments. The measured percentage in ^166^Ho count-density difference in the healthy-liver serves as an upper bound to the error in healthy-liver dosimetry, i.e., when all ^166^Ho activity resides within the healthy-liver compartment, the count-density offset due to poor scatter correction is negated as a result of the scaling process.

### Limitations and future developments

The presented value for the optimal k-factor is specific for the energy windows used in this work and includes the weight factor to account for the difference in window widths between the 81-keV and 118-keV windows. When different energy window settings are applied, care has to be taken to properly adapt, or re-evaluate, the required k-factor.

Whereas the phantom was filled with uniform activity, a more heterogeneous ^99m^Tc activity distribution will be encountered in patients. This may cause a relatively low ^99m^Tc activity in the healthy tissue to be classified as non-healthy tissue. Thus, the possibility to automatically segment the healthy-liver based on a threshold approach using ^99m^Tc images needs to be validated in clinical practice by comparing against manual segmentations of contrast enhanced CT images. In particular, the impact of adopting this method rather than the current manual approach for segmentation and registration has to be investigated with respect to dosimetry, time required to perform these tasks and inter-observer influence.

## Conclusion

Within the context of the ^166^Ho-^99m^Tc dual-isotope protocol, the impact of ^99m^Tc on reconstructed ^166^Ho count density in the healthy-liver could mostly be negated, provided an adequate k-factor for downscatter correction was applied during image reconstruction. The healthy-liver compartment of the phantom could accurately be segmented on the ^99m^Tc images using a thresholding method, irrespective of the amount of ^99m^Tc activity or in presence or not of ^166^Ho, and the healthy-liver compartment was recovered to 100% of its nominal volume (Sørensen–Dice index > 0.9).

Because additional scatter due to the presence of ^99m^Tc can effectively be corrected, the dual-isotope protocol can safely be applied without compromising ^166^Ho image quality. However, validation of the automatic segmentation method, and its effect on dosimetry, needs to be assessed in clinical practice.

## Data Availability

The datasets used and/or analyzed during the current study are available from the corresponding author on reasonable request.

## Abbreviations

^99m^Tc: Technetium-99 m
^166^Ho: Holmium-166
COV: Coefficient of variation
CT: Computed tomography
MAA: Macroaggregated albumin
*Q*_*H*_: Contrast recovery coefficient for the hot spheres
*Q*_*C*_: Contrast recovery coefficient for the cold spheres
S1: Sphere 1
S2: Sphere 2
SPECT: Single-photon emission computed tomography
VOI: Volume of interest
